# Non‐marine turtle plays important functional roles in Indonesian ecosystems

**DOI:** 10.1002/ece3.6487

**Published:** 2020-09-07

**Authors:** Nancy E. Karraker, Mirza Dikari Kusrini, Jessica R. Atutubo, Ryan M. Healey, Aini Yusratul

**Affiliations:** ^1^ Department of Natural Resources Science University of Rhode Island Kingston RI USA; ^2^ Department of Forest Conservation and Ecotourism Institut Pertanian Bogor Bogor Indonesia

**Keywords:** *Cuora amboinensis*, ecological roles, functional ecology, harvesting, illegal trade, over‐exploitation, seed dispersal, seed germination, trophic position, turtle

## Abstract

The Southeast Asian box turtle (*Cuora amboinensis*) is numerically the most important turtle exported from Indonesia. Listed as Vulnerable by the IUCN, this turtle is heavily harvested and exported for food and traditional medicine in China and for the pet trade primarily in the United States, Europe, and Japan. Despite its significance in global markets, relatively little is known about the species’ ecology or importance to ecosystems. We conducted our research in a national park in Sulawesi, Indonesia, and our objectives were to quantify trophic breadth, capacity for seed dispersal between aquatic and terrestrial ecosystems, and whether ingestion of seeds by *C. amboinensis* enhances germination. We obtained diet samples from 200 individual turtles and found that the species is omnivorous, exhibiting an ontogenetic shift from more carnivorous to more omnivorous. Both subadults and adults scavenged on other vertebrates. In a seed passage experiment, turtles passed seeds for 2‒9 days after ingestion. Radio‐tracked turtles moved, on average, about 35 m per day, indicating that seeds from ingested fruits, given seed passage durations, could be dispersed 70‒313 m from the parent tree and potentially between wetland and upland ecosystems. In a seed germination experiment, we found that ingestion of seeds by turtles enhanced germination, as compared with control seeds, for four of six plant species tested. Of these, two are common in the national park, making up a significant proportion of plant biomass in lowland swamp forest and around ephemeral pools in savanna, and are highly valued outside of the park for their lumber for construction of houses, furniture, and boats. Protection of *C. amboinensis* populations may be important for maintaining trophic linkages that benefit biodiversity, communities, and local economies.

## Graphical abstract

Despite its significance in global trade, relatively, little is known about the ecological roles of the Southeast Asian box turtle (*Cuora amboinensis*) in its ecosystems. At a study site in Indonesia, we determined that the species is omnivorous, disperses seeds between aquatic and terrestrial ecosystems, and enhances germination of four woody plant species through ingestion of fruit. Two of these trees are important for construction of houses, furniture, and boats and to the livelihoods of people in local communities.

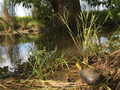

## 1 INTRODUCTION

Indonesia is a hotspot for turtle diversity (Buhlmann et al., 2009), with 25 native species of non‐marine turtles. Currently, 80% of Indonesia's non‐marine turtles are classified as Vulnerable, Endangered, or Critically Endangered by the International Union for the Conservation of Nature (IUCN, 2020), and for most, legal and illegal harvesting (Schoppe, 2009) and habitat loss associated with human population growth and a rapidly developing economy (Williams, 2013) threaten populations. Turtle populations continue to decline throughout Southeast Asia, including Indonesia; yet, we have limited understanding of their biology, especially ecology. This lack of information constrains conservation efforts (Shelmidine, Murphy, & Massarone, 2016; Wei, Gong, Shi, & Li, 2016), including captive breeding programs and initiatives to restore populations in the wild. Scientists have made repeated calls (Iskandar & Erdelen, 2006; Schoppe, 2009; Shen, Pike, & Du, 2010; Sung, Karraker, & Hau, 2013) for research on Asian turtles where viable populations still exist, in order to inform conservation efforts and to understand how population declines may impact other ecosystem constituents.

Non‐marine turtles play important functional roles in their ecosystems, yet the services they provide to their environments and to humans in those environments remain poorly studied (Lovich, Ennen, Agha, & Gibbons, 2018). Some evidence suggests that nonmarine turtles are important vectors for seed dispersal and enhance seed germination. Turtles swallow fruits whole, without crushing the seeds, and seeds of some plants that have passed through the guts of turtles exhibit increased germination success (Cobo & Andreu, 1988; Sung, Hau, & Karraker, 2016). Long passage duration in turtles, relative to other vertebrates, results in a weakening of the seed coat, increasing the probability and rate of germination, and allows seeds to be transported longer distances where they may have a higher likelihood of survival because of reduced competition with the parent plant and removal from the allelopathic zone beneath the parent plant (Herrera, 2002). Seed passage durations of four (Strong & Fragoso, 2006) to 14 days (Varela & Bucher, 2002) through the guts of some turtles indicate potential for long‐distance dispersal, contributing to germination success by reducing risks associated with seed predators searching beneath parent plants (Bjorndal, 1989). Turtles may be largely responsible for the dispersal and germination of some plants, such as the Galapagos tomato (*Lycopersicon esculentum*) by the Galapagos tortoise (*Geochelone nigra*) (Rick & Bowman, 1961). Further, low seed germination rates and population declines in endangered tambalacoque trees (*Sideroxylon* spp.) of Mauritius may be attributable to extinction of giant *Cylindraspis* tortoises which may have been necessary for germination of seeds (Iverson, 1987).

It has also been suggested that turtles may make important contributions to nutrient transfer between aquatic and terrestrial ecosystems. Aquatic and terrestrial ecosystems are linked by resource subsidies being transferred between them. Resource subsidies, including nutrients, propagules, and energy, that are contributed from one ecosystem to another (Richardson, Zhang, & Marczak, 2010) enhance the growth and development of organisms in the receiving ecosystem (Polis, Anderson, & Holt, 1997), thus contributing to the maintenance of overall biodiversity. Sheer densities of over 2000 turtles/ha (Cox & Marion, 1979; DeGregorio, Grosse, & Gibbons, 2012) and standing crop biomass of up to 877 kg/ha (Congdon, Greene, & Gibbons, 1986) of unexploited turtle populations in some ecosystems connotes an importance of these vertebrates (Iverson, 1982) that is largely understudied. Transfer of resources from one ecosystem to another by larger animals can replenish depleted nutrient stores and stimulate primary production (Vanni, 2002; Vanni, Flecker, Hood, & Headworth, 2002). Many species of aquatic turtles move from wetland to upland habitats to forage, nest, and estivate, thus transporting resources from aquatic to terrestrial habitats. To date, limited research has examined the contributions that freshwater turtles make to cross‐ecosystem resource flows, but the large quantities of plant and animal biomass consumed by some turtles (Coe, Bourn, & Swingland, 1979; Spencer, Thompson, & Hume, 1998), coupled with relatively large home ranges (Arvisais et al., 2002; Fachín‐Terán, Vogt, & Thorbjarnarson, 2006) and frequent movements between aquatic and terrestrial habitats (Arvisais et al., 2002; Semlitsch & Bodie, 2003), indicate that comparable relationships may exist.

Population declines can disrupt important ecological linkages, but it is not known how declines in freshwater turtle populations impact their ecosystems. The Southeast Asian box turtle (*Cuora amboinensis*) is numerically the most important turtle exported from Indonesia (Schoppe, 2009). Listed as Vulnerable by the IUCN (Asian Turtle Trade Working Group, 2000), Indonesia's populations of this turtle are heavily harvested for food and traditional medicine trades largely in Hong Kong and mainland China (Robinson, Griffiths, John, & Roberts, 2015; Schoppe & Das, 2011) and the pet trade in the United States, Japan, and several European countries (Auliya et al., 2016; Schlaepfer, Hoover, & Dodd, 2005). As with many other turtles, delayed sexual maturity and small clutch sizes limit the capacity of populations to recover from harvesting. Although populations are probably declining in Indonesia, remaining viable populations present an increasingly rare opportunity in Asia to study the species’ ecological roles and understand how population losses may affect ecosystems. The objectives of our study were to identify the ecological roles of one of the world's most heavily traded turtles by: (a) characterizing trophic breadth of *C. amboinensis* through ontogeny, (b) determining capacity for seed dispersal, and (3) quantifying contributions of this turtle to seed germination.

## 2 METHODS

### 2.1 Study area and animal

**FIGURE 1:**
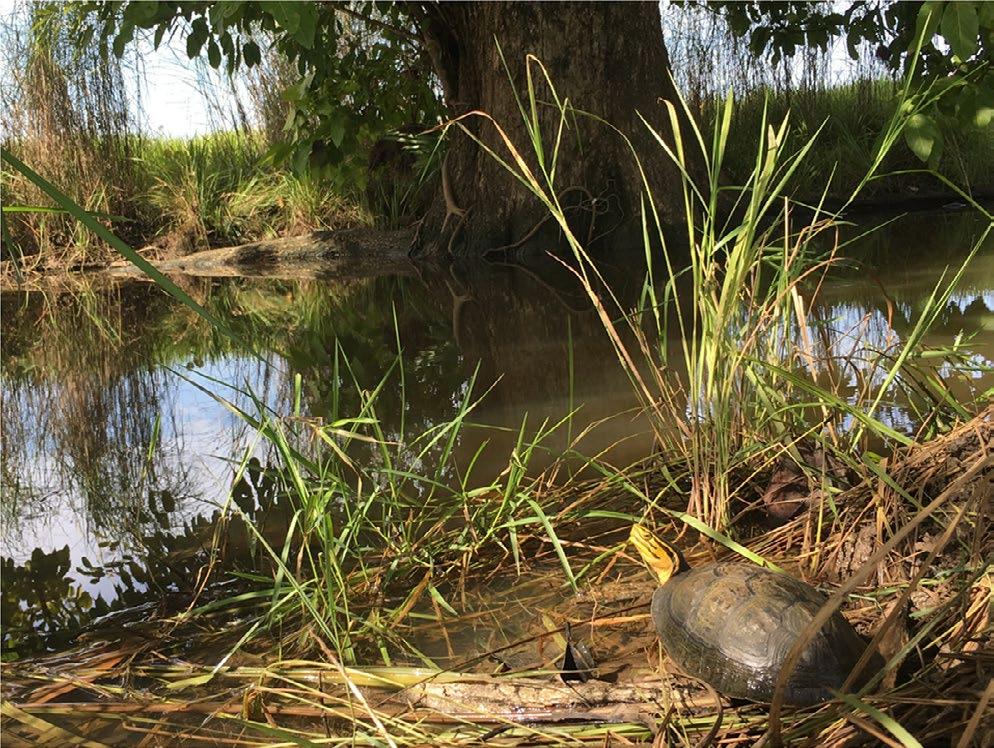
Adult Southeast Asian box turtle (*Cuora amboinensis*) near an ephemeral wetland in savanna, Rawa Aopa Watumohai National Park, South East Sulawesi, Indonesia. Photograph was taken by N. Karraker on 25 April 2018

We conducted this research from December 2017 to April 2018 as part of a broader project evaluating populations, movements, and ecological roles of *C. amboinensis* in and around Rawa Aopa Watomohai National Park in the province of South East Sulawesi, Sulawesi, Indonesia. Habitats where turtles were found included lowland swamp forest, streams, and ephemeral wetlands inside the national park, and ponds and impounded wetlands in agricultural areas outside of the national park (Figure 1).

*Cuora amboinensis* ranges from Bangladesh through Southeast Asia to Indonesia, where individuals inhabit lowland wetlands (Schoppe & Das, 2011). In Indonesia, the species is broadly distributed throughout most of the archipelago to the eastern provinces of Maluku and North Maluku and historically has been locally abundant. Individuals held in captivity reach reproductive maturity at 6–9 years and females lay 1–4 eggs (summarized in Schoppe & Das, 2011). Adults are sexually dimorphic, wherein males usually have a concave plastron and a thicker, longer tail; females generally have a flat plastron and have a thinner, shorter tail. This is the only species of nonmarine turtle in Southeast Sulawesi.

### 2.2 Trophic breadth

To characterize trophic breadth of *C. amboinensis*, we trapped turtles inside and outside of the national park using collapsible turtle traps measuring 60 × 30 cm, with 13 cm funnel‐shaped openings on each end. Traps were baited with about 25 g of sardines held inside a cylindrical 12 cm × 2.6 cm plastic tube with a screw top. We drilled holes into the sides of each tube, thereby allowing turtles to smell the bait but not eat it. Traps also contained a flotation device so that they would not sink, permitting turtles access to the water surface to breathe. This species readily defecates upon handling, so without removing traps from the water, captured turtles were quickly removed from traps and placed into plastic buckets where they were held for 30 min. If defection occurred during the 30‐min period, we collected the feces into a plastic container and made a small notch with a triangular file on the right #6 marginal scute of the turtle to indicate that a diet sample had been taken. Only one diet sample was collected from each turtle. We weighed each turtle and measured carapace and plastron length and width. Because of distinct wet and dry seasons in the region, turtles can be aged by counting annuli on their scutes. We aged turtles up to 15 years; after 15 years, annuli begin to grow over each other and age cannot be reliably determined. Turtles aged six years or older that exhibited sexually dimorphic traits were considered to be adults (Schoppe & Das, 2011).

In a makeshift laboratory, we sorted fecal samples in a large dish and all seeds, insect parts, vertebrate parts, and other identifiable diet items were separated from other material. Seeds, insects, and vertebrates were identified to the lowest taxonomic level possible using a field microscope (Nikon Model 7314, Nikon Corporation) at 20× magnification. For animal matter, we counted numbers of individuals of each taxonomic group in a sample, and for fruit, we counted the number of seeds or stems (of herbaceous plants) of each taxonomic group.

To identify plant species consumed by *C. amboinensis*, we collected all fruits and seeds that we found near turtle habitats in the national park throughout the study period. Leaves, fruits, and seeds of each plant taxon were photographed, measured, and identified to the lowest taxonomic level possible using published references from the region and with assistance from local, regional, and international plant experts. A subset of seeds of each plant taxon was stored as a reference collection to aid in identifying seeds in turtle feces.

We calculated frequency of occurrence as the percentage of individuals in each age/sex class that had consumed a particular diet item (Bowen, 1983) and for each trophic level. We quantified niche breadth using the Shannon Index and the evenness Index. We determined the proportion of subadult and adult turtles that had scavenged other vertebrates.

On a monthly basis, we documented the presence of the most commonly eaten fruits on the ground around turtle habitats to establish fruiting periods during our study and understand the importance of particular fruits to the diet of this turtle.

### 2.3 Capacity for seed dispersal

To quantify capacity for seed dispersal and nutrient transport by *C. amboinensis*, we estimated numbers of fruits and seeds eaten, passage times for seeds through the digestive tract, and determined distances that turtles moved. As it is believed that subadult turtles are less likely to leave water because their small size puts them at greater risk of predation in terrestrial habitats, we focused this part of the study on adult turtles, which are more likely to move between aquatic and terrestrial habitats.

To determine duration of seed passage through the digestive system of *C. amboinensis*, we used *Cayratia trifolia* (Vitaceae), the fruits of which belong to one of the four most frequently ingested plant taxa. We captured male and female turtles from areas in which we did not observe *C. trifolia*, which grows as a vine and is obvious around wetlands. Each turtle was held for 30 min in the field, as described above, to obtain a diet sample and then was transported back to the research station. We placed turtles in individual plastic boxes measuring (L × W × H) 46 × 31 × 35 cm containing 2 cm of well water and a brick so that turtles could get out of the water. We placed plastic containers in an outside shelter that received ambient light but no direct sunlight. Turtles were held and not fed for 48 hr to evacuate their digestive tracts. We checked turtles every 12 hr and documented whether they had defecated. Water was changed every 24 hr throughout the experiment, unless the turtle had defecated and then it was changed at the intervening 12‐hr check. After 48 hr, we provided turtles with 10‒11 fruits (20–25 g) of *C. trifolia*. The fruits of this plant generally contain 1‒2 large seeds, making them obvious in fecal samples. We checked turtles every 12 hr and documented number of fruits consumed. If defecation had occurred the box was emptied, contents were examined for seeds, and the number of seeds defecated was documented. We terminated each trial 10 days after a turtle consumed the fruit, or three days if the turtle did not consume the fruit, and released the turtle where it was originally captured. Each turtle was used for only one experimental trial and we made a small notch in right marginal scute #7 prior to release to prevent reuse of turtles in the experiment. We summarized number of seeds passed, mean days to first seed passed, and mean days to last seed passed.

To determine the number of seeds per fruit, we collected mature fruit from the forest floor of some of the most commonly eaten species, including *Artocarpus* cf. *teijsmannii* (Moraceae, *n* = 14), *C. trifolia* (*n* = 8), *Dillenia serrata* (Dilleniaceae, *n* = 8), and *Vitex cofassus* (Lamiaceae, *n* = 13). We removed the pulp from each fruit, and extracted and counted the seeds. By comparing the number of seeds of particular plants in each diet sample and the average numbers of seeds in the fruit of those plants, we estimated how many individual fruits a turtle had consumed prior to capture, and thus how many seeds were likely to be dispersed. Our methods did not enable us to determine if a turtle had defecated prior to or during capture in the trap and what might remain undigested in the stomach. Thus, our estimates for numbers of fruits eaten should be considered conservative. We were unable to determine the number of seeds per fruit for two other important species, *Ficus* spp. (Moraceae) and *Neolamarckia cadamba* (Rubiaceae), as their fruits contain thousands of minute, delicate seeds that are nearly impossible to separate from the surrounding pulp.

We captured 10 turtles (five females, five males) for radio telemetry in February 2018 and tracked them through April 2018. Four turtles originated in savanna wetlands and six originated in forested swamp in the national park. We attached a Holohil RI‐2B radio transmitter to the rear right portion of the carapace of each turtle using marine epoxy. For the original capture and each subsequent relocation, we recorded geographic coordinates (Universal Transverse Mercator; North American Datum of 1983) using a global positioning system (Garmin Oregon, 450). After radio attachment and data collection, we released turtles where they were originally found. Turtles with radios were tracked every three days with a receiver (Advanced Telemetry Systems, Model R410) and a three‐element Yagi antenna. When a turtle was relocated, we remained at least 0.5 m away to limit disturbance to the turtle as we collected data for a companion movements study. We calculated average daily movement distance for each turtle.

### 2.4 Seed germination

To determine if seed germination was enhanced following ingestion by a turtle, we conducted germination trials of ingested seeds from fecal samples compared with control seeds collected from the forest. Control seeds were collected by finding mature fruit on the forest floor and removing the seeds from the pulp. Seeds of both types were rinsed with well water. Seeds were placed in germination bags which consisted of a paper towel measuring 22 × 11 cm with an 11 × 11 cm sheet of clear plastic beneath it and a clip holding the edge of the paper towel onto the plastic. Ten control or ingested seeds of a given genus or species were placed on the half of the paper towel seated on the plastic. The other half of the paper towel was saturated with well water and then folded over on top of the seeds on the plastic sheet. The plastic sheet with saturated paper towel containing the seeds was slid into a clear zip‐top plastic bag (17 × 15 cm). We inflated germination bags by blowing into them so that the upper surface of the plastic bag was not resting on the top of the paper towel. Germination bags were randomly positioned in a box measuring 195 × 189 × 12 cm and placed on a table beneath a roof outside. The bags received ambient light but none received direct sunlight at any time of the day. Each morning, we inspected seeds in germination bags. Germination of seeds of *A. teijsmannii*, *C. trifolia*, *D. serrata*, and *V. cofassus* could be seen unaided, but we examined seeds of *Ficus* spp. and *N. cadamba* using a microscope at 20× magnification, because of their small size. About 4 ml of well water was added to the upper surface of the paper towel every three days or as needed to keep seeds moist.

For each of six plant species, we conducted germination trials on seven replicates of seeds collected from the forest (controls) and seven to 11 replicates of seeds that had been ingested by turtles. We documented date of germination for each seed and removed germinated seeds from the bag to prevent damage to the paper towel by the cotyledon. Trials were terminated after 30 days for each germination bag because pilot trials indicated that all of our target species were capable of germination within one to two weeks, fruiting periods varied between species necessitating seed collection when available, and because of limited space in the germination box. We compared germinability (final percent germinated) between ingested and control seeds for each plant species using a general linear model with a binomial distribution and a logit‐link function. We compared germination rate (days to germination) for seeds ingested by turtles and control seeds for each plant species using a general linear model with a Poisson distribution. Statistical analyses were conducted in SAS (SAS Institute, version 9.4).

## 3 RESULTS

### 3.1 Trophic breadth

We obtained diet samples from 200 adult and subadult *C. amboinensis* from inside and outside the national park. Of these, 23 contained only unidentifiable plant material and were not included in the analysis. Of the remaining samples (*n* = 177), 42% were from subadults, 37% were from adult males, and 21% were from adult females. We documented invertebrates more frequently in the diets of subadults and fruits more frequently in the diets of adults (Table 1). Based on the Shannon and evenness indices, subadult turtles had a wider niche breadth than adults, and females had a wider niche breadth than males (Table 1).

**Table 1:** Frequency of occurrence of food items, as a percent of individuals containing a particular item, in the diets of adult and subadult Southeast Asian box turtles (*Cuora amboinensis*) in South East Sulawesi, Indonesia from January to April 2018

Diet item, Trophic level consumption	Frequency of occurrence (%)			
	Subadult	Male	Female	Adult
Fruit	16	92	69	82
Grass/sedge/rush	11	13	22	16
**Primary consumption**	**25**	**95**	**81**	**88**
Earthworm	0	2	0	1
Arachnid	5	2	3	2
Insect	76	48	44	44
Bivalve	32	2	8	4
Gastropod	23	6	31	15
Crustacean	9	3	8	5
Fish	3	0	0	0
Snake	1	11	0	7
Lizard	1	0	0	0
Bird	0	5	0	3
Mammal	4	3	8	5
**Secondary consumption**	**89**	**64**	**75**	**67**
*Shannon index H′*	2.1598	1.6564	2.0309	1.9260
*Evenness index*	0.9007	0.6908	0.9767	0.8032
*Sample size*	75	66	36	102

Note

Frequency of occurrence for each trophic level was calculated as the percent of individuals containing any diet item within that trophic level.

Fruit appeared in the diets of 82% of adults. For all adults that had eaten fruit, *Artocarpus* cf. *teijsmannii* (37%), *N. cadamba* (35%), and *C. trifolia* (19%) occurred most frequently in the diet. *Ficus* spp. (14%), *D. serrata* (6%), *V. cofassus* (4%), *Lagerstroemia* cf. *speciosa* (1%), and the seeds of two unidentified woody plants (5%, 1%) also appeared in the diet of adults. Fruit occurred in the diet of 12% of subadults. Of the subadults that had eaten fruit, *C. trifolia* (42%), *N. cadamba* (17%), *V. cofassus* (17%), and *Ficus* spp. (17%) occurred most frequently. *Artocarpus* cf. *teijsmannii* (8%) was the only other woody plant species found in the diet of subadults. Across both age classes and sexes, we documented that turtles consumed the fruits of 9‒15 species of woody plants. Six *Ficus* spp. occurred in the area, and we were not able to differentiate the seeds among species.

We documented invertebrates in diet samples of 62% of adults (Table 1). Insects appeared in 44% of samples, and most frequent were Coleoptera (49%), Isoptera (19%), and Diptera (17%). Invertebrates occurred in diet samples of 88% of subadults (Table 1). Insects were present in 76% of samples from subadults, and the taxa with the highest frequency of occurrence were Coleoptera (53%), Diptera (25%), and Odonata (18%). We were unable to determine if invertebrates had been consumed live or had been scavenged.

Fifteen percent of adults had consumed vertebrates, including snakes, birds, and mammals, and 12% percent of subadult turtles had fed on fish, snakes, lizards, and mammals (Table 1). As this turtle is fairly slow‐moving, we expect that turtles had scavenged the remains of vertebrates.

Timing and duration of fruiting, and thus availability of fruit of the most important species in the diet varied considerably. *Artocarpus* cf. *teijsmannii* and *C. trifolia* produced mature fruit continuously from January through April. We also observed mature fruits on the ground continuously for *Ficus* spp., but as we could not differentiate taxa within the genus we could not delineate the fruiting periods for individual species. We first observed mature fruits of *V. cofassus* beginning in the third week of February and continuously through April. Mature fruits of *N. cadamba* were first observed in the third week of March and this species continued fruiting through April. Finally, *D. serrata* produced mature fruit for a brief period of one to two weeks at the beginning of January and again in the middle of April, which continued through our departure at the end of April.

### 3.2 Capacity for seed dispersal

We examined seed passage in seven turtles, including four females and three males, that consumed fruit of *C. trifolia*. Males passed more seeds than females (Table 2) over the 10‐day period. Mean days to passing of the first seed was the same for males and females, but males passed seeds later in the experiment than did females (Table 2).

**Table 2:** Number of seeds passed and duration of seed passage for Southeast Asian box turtles (*Cuora amboinensis*) fed fruits of *Cayratia trifolia* and observed for 10 days in South East Sulawesi, Indonesia in 2018

Sex	*n*	Mean number of seeds passed		Mean days to first seed passed		Mean days to last seed passed	
		Mean (SE)	Range	Mean (SE)	Range	Mean (SE)	Range
Male	3	19.7 (5.7)	13‒31	3.3 (0.7)	2‒4	7.3 (0.9)	6‒9
Female	4	12.3 (7.7)	0‒33	3.3 (2.0)	2‒9	4.0 (1.9)	3‒9
Both sexes	7	15.4 (4.9)	0‒33	3.3 (1.1)	2‒9	5.4 (1.3)	3‒9

For the most common species of plants in the diet, we determined the number of seeds consumed by each adult turtle and that thus were possible of being dispersed from beneath the parent tree to another location. On average (±*SE*), adult turtles had consumed 4.5 seeds (±1.1 seeds, range 1‒25 seeds, *n* = 31) of *A*. cf. *teijsmannii*, 8.3 seeds (±2.1 seeds, range 1‒30 seeds, *n* = 15) of *C. trifolia*, 25.2 seeds (±7.9 seeds, range 7‒50 seeds, *n* = 5) of *D. serrata*, and 1.5 seeds (±0.5 seeds, range 1‒2 seeds, *n* = 2) of *V. cofassus*. Minute seeds of *N. cadamba* and *Ficus* spp. in fecal samples numbered in the hundreds, and we were not able to ensure accurate counts.

Radio‐tracked turtles moved a mean distance (±*SE*) of 34.8 m/day (±4.5 m/day; range 0‒173.4 m/day). These data on turtle movements coupled with gut passage times of 2‒9 days indicate that fecal contents, including seeds and nutrients, may be dispersed, on average, 70‒313 m from locations where plants and animals were consumed.

### 3.3 Seed germination

**FIGURE 2:**
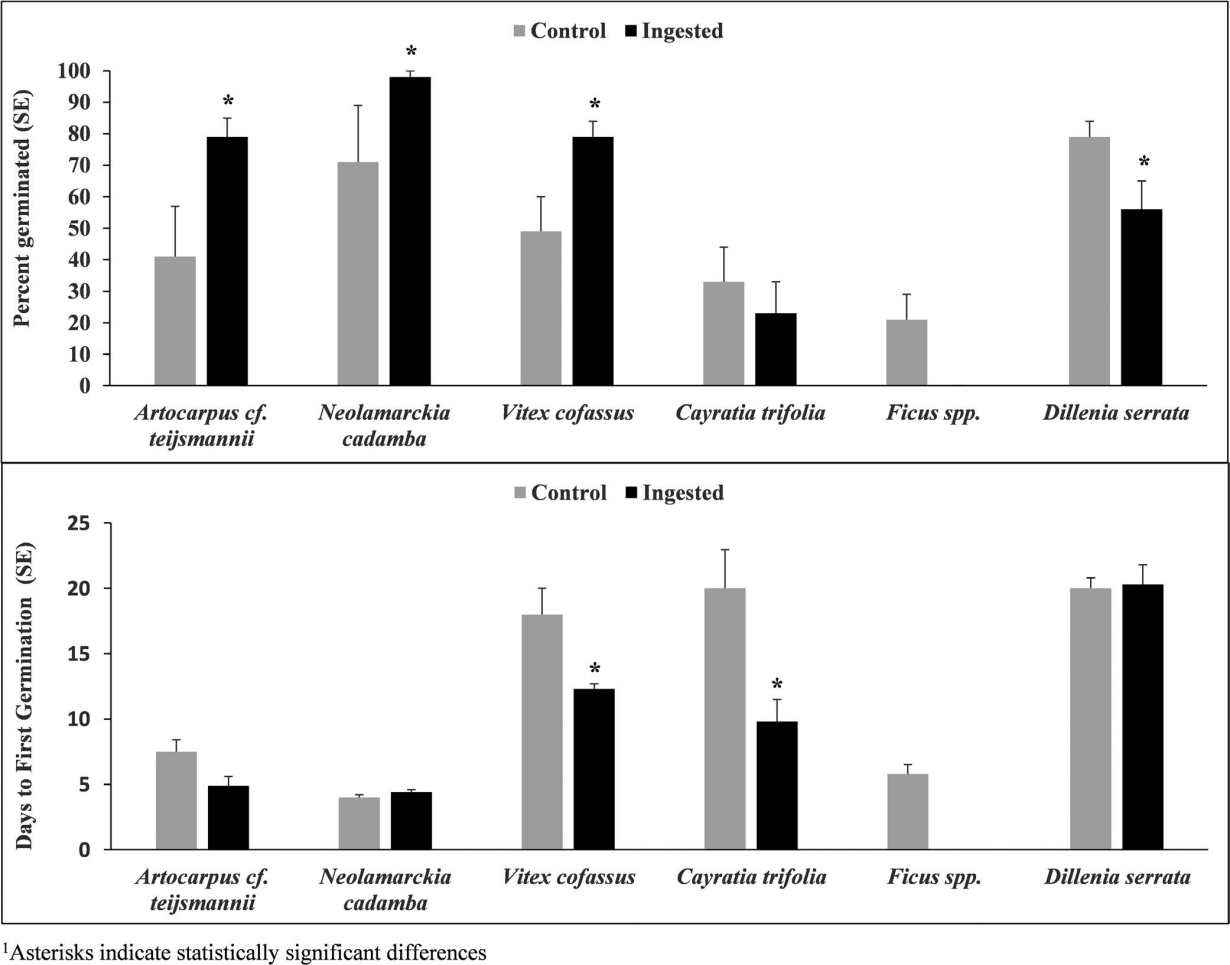
Mean percent germinated (±*SE*) and mean germination rate (±*SE*) of seeds from the forest (control) and those ingested by Southeast Asian box turtles (*Cuora amboinensis*) in South East Sulawesi, Indonesia in 2018

We compared germinability between seeds ingested by turtles and control seeds collected from the forest floor for *A*. cf. *teijsmannii*, *N. cadamba*, *C. trifolia*, *D. serrata, Ficus* sp., and *V. cofassus* (Figure 2). For seeds that had been ingested by turtles, germinability was 38% higher for *A*. cf. *teijsmanni* (*z* = −4.96, *p* < .0001), 30% higher for *V. cofassus* (*z* = −3.76, *p* = .0002), and 27% higher for *N. cadamba* (*z* = −3.60, *p* = .0003), compared with control seeds. Germinability for seeds ingested by turtles was 23% lower for *D. serrata* (*z* = 2.83, *p* = .0046), and ingestion by turtles did not significantly influence germinability of seeds of *C. trifolia* (*z* = 1.31, *p* = .1895) or *Ficus* spp. (*z* = 1.48, *p* = .1392).

We compared germination rates for ingested and control seeds of the same six plants (Figure 2). Seeds ingested by turtles germinated in 50% of the time for *C. trifolia* (*z* = 4.46, *p* < .0001) and in 67% of the time for *V. cofassus* (*z* = 2.52, *p* = .0117), as compared with control seeds. Germination rate between seeds ingested by turtles and control seeds were not statistically different for *A*. cf. *teijsmanni* (*z* = 1.64, *p* = .1012), *N. cadamba* (*z* = −0.35, *p* = .7238), or *D. serrata* (*z* = 0.08, *p* = .936). No *Ficus* spp. seeds ingested by turtles germinated for comparison with control seeds.

## 4 DISCUSSION

### 4.1 Trophic breadth

*Cuora amboinensis* are generalist omnivores that feed on a wide range of plants and animals and scavenge on vertebrates. Fruits of at least nine species of woody plants occur in the diet of *C. amboinensis*. Given the relatively short duration of our study and that seeds of six *Ficus* spp. that occur in the national park were indistinguishable among species, this number is certainly higher. *Artocarpus* cf. *teijsmannii* and *V. cofassus* grow within lowland swamp forest. *Neolamarckia cadamba* is found on the edges of ephemeral wetlands in savanna. The fruiting vine, *C. trifolia*, appears to be wetland associated and was found growing on trees in both swamp forest and on the edges of ephemeral wetlands. Turtles in lowland swamps and ephemeral wetlands feed on fruits that have fallen into the water. In contrast. *D. serrata* and *Ficus* spp. are upland forest trees and turtles feed on these fruits on the forest floor.

Notably, inside the national park, seeds of *A*. cf. *teijsmannii* occurred in the diet of 40% of adult turtles that had consumed fruit. This large tree is abundant in lowland swamp forest and produced large quantities of fruit continuously during our study. Seeds of another large tree, *N. cadamba*, appeared in the diet of 32% of adult turtles inside the national park that had consumed fruit. This large tree is often the only species of tree that grows at the edges of ephemeral wetlands in savanna. *Neolamarckia cadamba* produces substantial quantities of large fruit and fruit matured during the latter one‐third of our study period. Outside of the park, sampled wetlands were surrounded largely by agricultural trees, including sago palm (*Cycas revoluta*), banana (*Musa* spp.), and coconut (*Cocos nucifera*), none of which is a source of food for *C. amboinensis*. *Neolamarckia cadamba* grew near one wetland outside of the park and 67% of adult turtles had consumed its fruit. The vine *C. trifolia* grew around the same wetland and its seeds appeared in the diet of 100% of adults captured there.

We documented an ontogenetic shift in diet, with subadult turtles consuming a higher proportion of invertebrates and vertebrates, and adults becoming more omnivorous. Similar ontogenetic changes in diet have been documented in other species of semi‐aquatic turtles (Clark & Gibbons, 1969; Sung et al., 2016) and, in the case of *C. amboinensis*, this shift is probably related in part to gape and bite force limitations in younger individuals. Many of the fruits we documented around wetland habitats were too large to have been swallowed whole by smaller turtles and a tough exocarp may limit access to the fleshy pulp inside.

Both subadults and adults are secondary consumers feeding on insects, mollusks, crustaceans, and other invertebrate groups. Notably, both age classes appear to play a role in energy flow through these ecosystems with 12% of subadults and 15% of adults in our study having scavenged on other vertebrates. We determined that a turtle had scavenged on vertebrates when we found fish scales, feathers, bones, or skin in its feces, but we were unable to determine if invertebrates were consumed live or scavenged. Thus, the overall importance of scavenging to this species is likely underestimated by our data.

In addition to their roles as predators and scavengers in aquatic and terrestrial habitats and moving nutrients between ecosystems, *C. amboinenis* are likely prey for birds and mammals in our study area. Although we did not document instances of predation, *C. amboinensis*, and particularly eggs and hatchlings, have been documented as prey of crocodiles, monitor lizards, wetland birds, and mid‐sized mammals, such as civets (Moll & Moll, 2004; Stanner, 2010). Given that this turtle has a hinged plastron, or lower shell, and can completely enclose its limbs and head within its shell, predation risk is probably relatively low for adults.

### 4.2 Capacity for seed dispersal

Frequency of fruit in the diets of adults, regular movements of turtles between wetland and upland, and seed passage durations of at least 2‒9 days indicate that *C. amboinensis* is capable of transporting seeds away from parent trees where there is an increased likelihood of germination. Movements of adult *C. amboinensis* are triggered in part by the ephemerality of the wetlands they inhabit. Lowland swamp forest is flooded by monsoonal rains typically from January to July each year, but repeated flooding and drying occurs between major rain events. Adult turtles move into swamps after flooding, where they will feed on fruits on the swamp bottom. As swamps dry down and become clusters of large puddles, turtles often move back into the forest. Similarly, ephemeral pools in savanna fill around January and generally retain water until July, although depth varies significantly between major rain storms. Turtles feed on fruits on the pool bottom and venture to forest patches nearby where they will spend days feeding on fruits on the forest floor. Movements of seeds from swamp or pool bottoms to their edges and dispersal of seeds of upland trees as turtles move between forest and savanna may increase the likelihood that those seeds will germinate.

Seed dispersal by animals provides a number of benefits to plants including removal of seeds from inhospitable conditions near the parent tree and transport to perhaps more suitable conditions at a seed's final destination (Herrera, 2002). Many species of plants produce allelopathic compounds that become infused in the soil beneath the plant, inhibiting germination of seeds of other plants as well as germination of their own seeds. Unless a seed can disperse beyond the allelopathic and water‐ and light‐limited zone beneath its parent tree, an area that is targeted by seed predators (Kuprewicz, 2013), germination and growth to maturity are improbable. The mechanism by which seeds leave the parent plant may influence seed viability. Seed dispersing animals, as in some species of primates (Ungar, 1995), may feed on unripe fruit thereby ingesting immature seeds that are incapable of germinating after defecation. In contrast, seeds in ripe fruit that has fallen to the ground will be mature and have a higher likelihood of germination following ingestion by turtles and dispersal.

Researchers (Stiles, 2000) have suggested that few reptiles are important seed dispersers, and that these roles are limited to iguanid lizards and terrestrial turtles. However, such statements are more a reflection of limited work on reptiles, and particularly on semi‐aquatic turtles such as *C. amboinensis*, relative to birds and mammals. The contributions of turtles to seed dispersal has been examined most intensively in completely terrestrial species, such as the tortoises (Testudinidae) of the Americas and box turtles (Emydidae) of North America. Importance of fruit in the diet, relatively large home ranges, seed retention times, and germinability of ingested seeds (Strong & Fragoso, 2006) suggest that tortoises may be important seed dispersers in some ecosystems. In contrast, highly aquatic turtles may consume fruit but may not disperse seeds to places were germination is likely. For example, the big‐headed turtle (*Platysternon megacephalum*) consumes the fruits of two species of plants that fall into high gradient streams where the seeds have a low probability of germination (Sung et al., 2016). However, ingestion of fruits by these turtles probably does not lead to increased germination of the seeds. As *P. megacaphalum* are highly aquatic and rarely leave the perennial streams they inhabit (Sung, Hau, & Karraker, 2015), most seeds are likely defecated back into the stream. Semi‐aquatic species, such as *C. amboinensis*, may aid in propagating both wetland‐ and upland‐associated plant species because of their ecologically dynamic life histories in which they move among aquatic habitats and between aquatic and terrestrial habitats.

### 4.3 Seed germination

Ingestion by animals may enhance seed germination by increasing the percentage of seeds that germinate (germinability) or increasing the germination rate after a seed has been dispersed. Ingestion by *C. amboinensis* appears to enhance germination of seeds in four of six plants we studied: *A*. cf. *teijsmannii* (germinability)*, N. cadamba* (germinability), *V. cofassus* (germinability + germination rate), and *C. trifolia* (germination rate). Ingestion did not influence germination of seeds of *D. serrata* or *Ficus* spp. It is likely that scarification of the seed coat occurs through physical and chemical processes during digestion by *C. amboinensis* that increases germinability and/or germination rate, as has been indicated for some birds (reviewed in Traveset, 1998). Processing of fruit by animals may also impact viability and potential for germination. Animals that chew, such as rodents, often damage seeds (Hulme, 2002), in contrast with other taxa, such as birds (Wheelwright, 1985) and turtles (Moskovits & Bjorndal, 1990), that swallow fruit whole or in large portions.

Ingestion by *C. amboinensis* enhanced germinability of seeds of two of the most important trees in the national park, *A*. cf. *teijsmannii* and *N. cadamba*, by greater than 25%. *Artocarpus* cf. *teijsmannii* is distributed throughout Malaysia, Indonesia, and Papua New Guinea (Williams et al., 2017), and is one of the most common trees in lowland swamp forest in the national park. It grows to 45 m in height and up to 80 cm in diameter and appears to represent a significant proportion of the plant biomass in the lowland swamp forest ecosystem. Fruit of this tree is important to booted macaques (*Macaca ochreata*; Bismark, Gunawan, Tikupadang, & Iskandar, 1996), a primate that is endemic to southeast Sulawesi and listed by the IUCN as Vulnerable (Supriatna, 2008). We observed birds and booted macaques feeding in the trees and noted seeds of this tree in civet feces along the trails, indicating that these groups may also aid in seed dispersal and germination.

*Neolamarckia cadamba* ranges from India to China and through Southeast Asia to Indonesia and Australia. This tree grows to 45 m in height and up to 160 cm in diameter, and in the national park, this is the most common tree, and often the only species of tree, growing around ephemeral wetlands in savanna. Many of these isolated ephemeral wetlands in which we found turtles were located hundreds of meters from the nearest forest patch or nearest other ephemeral wetland. Given the capacity of *C. amboinensis* to move long distances, as we documented by radio telemetry, we expect that turtles move back across the savanna to forest patches on an annual basis when these ephemeral pools dry, with isolated pools in the savanna perhaps serving as stepping stones of suitable habitat. The importance of *N. cadamba* in the diet and movements of turtles between isolated ephemeral wetlands and forest patches suggests that these turtles may be playing an important role in propagation of *N. cadamba* in the savanna and potentially contribute to plant succession as clusters of trees begin to grow around these wetlands. Forest‐dwelling mammals are unlikely to cross large expanses of savanna, so we expect that *C. amboinensis* and birds are the most likely vertebrate groups aiding in propagation of *N. cadamba* at these wetlands in the savanna. Notably, we documented lower densities of turtles outside of the national park (N.E. Karraker, unpublished data) and observed *N. cadamba* at only one wetland outside of the park.

### 4.4 Conservation implications

Unimpacted populations of *Cuora amboinensis* exhibit relatively high local abundances, in which adults make regular movements between aquatic and terrestrial habitats and between lowland swamp forest and savanna ecosystems consuming a diverse array of animals and plants. As the only species of nonmarine turtle in southeast Sulawesi, *C. amboinensis* appear to play important roles moving nutrients and seeds between habitats and ecosystems and aiding in seed germination for two of the most significant tree species, *A*. cf. *teijsmannii* and *N. cadamba*, in the national park. Movements of these turtles hundreds of meters between isolated ephemeral wetlands in savanna and forest patches, the importance of the fruits of *N. cadamba* in their diet, and increases in germination success of *N. cadamba* seeds following ingestion, suggest that *C. amboinensis* may be aiding in propagation of *N. cadamba* in the savanna and, perhaps, plant succession there. Although we do not have comparative data for birds or mammals, we expect that the contributions by *C. amboinensis* are important and warrant further study and consideration.

Ecological data, such as those we have collected for *C. amboinensis*, are becoming increasingly essential for informing emergency triage situations following law enforcement confiscations of large numbers of turtles, managing longer‐term rehabilitation and care of confiscated turtles, and guiding husbandry of captive assurance colonies established for critically endangered turtles. Over the past decade, we have witnessed numerous news reports of confiscations of turtles illegally collected and destined for trade, numbering >1,000 in Myanmar, >4,000 in the Philippines, and nearly 11,000 in Madagascar, along with the confiscation of smaller numbers of individuals en route to or within markets (Leupen, 2018; Morgan, 2018). Larger confiscations of turtles, often with compromised health, have necessitated rapid establishment of triage facilities and longer‐term rehabilitation centers. A lack of ecological information, particularly on the diets of these species, likely slows recovery in captivity and lengthens the time before turtles can be repatriated. For example, understanding the timing of ontogenetic (Bouchard & Bjorndal, 2005) or seasonal (Del Vecchio, Burke, Rugiero, Capula, & Luiselli, 2011) shifts in diet can aid veterinary practitioners and other staff at these care centers in determining the optimal diet for individuals, thereby accelerating the pace of rehabilitation or maintaining turtles in good health in captive assurance colonies.

Finally, the conservation implications of our results extend also to Indonesian communities reliant on *A*. cf. *teijsmannii* and *N. cadamba*. Both trees are important for livelihoods and local economies in Indonesia that rely on lumber and wood products for construction of furniture, homes, and boats (Khairil, 2017; Krisnawati, Kallio, & Kanninen, 2011). Fruits of both species are eaten by humans and fruit of *A*. cf. *teijsmannii* is noted for an array of medicinal qualities (Achmad et al., 2002). Conservation of *C. amboinensis* will aid in maintaining the integrity of trophic linkages in these ecosystems, which also benefits Indonesian communities. We urge policymakers and land managers to recognize the importance of this species to sustaining the complexity of food webs and for its contributions to local economies and livelihoods.

## CONFLICT OF INTEREST

The authors declare not conflicts of interest.

## AUTHOR CONTRIBUTION

**Nancy Karraker:** Conceptualization (equal); Data curation (lead); Formal analysis (lead); Funding acquisition (lead); Investigation (lead); Methodology (lead); Project administration (equal); Resources (lead); Software (lead); Supervision (lead); Validation (lead); Visualization (lead); Writing‐original draft (lead); Writing‐review & editing (lead). **Mirza Dikari Kusrini:** Conceptualization (equal); Data curation (supporting); Formal analysis (supporting); Funding acquisition (supporting); Investigation (supporting); Methodology (supporting); Project administration (equal); Resources (supporting); Software (supporting); Supervision (supporting); Validation (supporting); Visualization (supporting); Writing‐original draft (supporting); Writing‐review & editing (supporting). **Jessica Atutubo:** Conceptualization (supporting); Data curation (supporting); Formal analysis (supporting); Funding acquisition (supporting); Investigation (supporting); Methodology (supporting); Project administration (supporting); Resources (supporting); Software (supporting); Supervision (supporting); Validation (supporting); Visualization (supporting); Writing‐original draft (supporting); Writing‐review & editing (supporting). **Ryan Healey:** Conceptualization (supporting); Data curation (supporting); Formal analysis (supporting); Funding acquisition (supporting); Investigation (supporting); Methodology (supporting); Project administration (supporting); Resources (supporting); Software (supporting); Supervision (supporting); Validation (supporting); Visualization (supporting); Writing‐original draft (supporting); Writing‐review & editing (supporting). **Aini Yusratul:** Conceptualization (supporting); Data curation (supporting); Formal analysis (supporting); Funding acquisition (supporting); Investigation (supporting); Methodology (supporting); Project administration (supporting); Resources (supporting); Software (supporting); Supervision (supporting); Validation (supporting); Visualization (supporting); Writing‐original draft (supporting); Writing‐review & editing (supporting).

## Data Availability

Data are available at: https://doi.org/10.6084/m9.figshare.1188814.
